# Creating a learning health system to include environmental determinants of health: The GroundsWell experience

**DOI:** 10.1002/lrh2.10461

**Published:** 2024-10-10

**Authors:** Sarah E. Rodgers, Rebecca S. Geary, Roberto Villegas‐Diaz, Iain E. Buchan, Hannah Burnett, Tom Clemens, Rebecca Crook, Helen Duckworth, Mark Alan Green, Elly King, Wenjing Zhang, Oliver Butters

**Affiliations:** ^1^ Public Health, Policy & Systems University of Liverpool Liverpool UK; ^2^ School of Geosciences, Institute of Geography University of Edinburgh Edinburgh UK; ^3^ NHS Arden & Great East Midlands Commissioning Support Unit Leicester UK; ^4^ Geography & Planning, Roxby Building University of Liverpool Liverpool UK

**Keywords:** data privacy, household record linkage, public health learning system, public health research, record linkage complex systems

## Abstract

**Introduction:**

Policies aiming to prevent ill health and reduce health inequalities need to consider the full complexity of health systems, including environmental determinants. A learning health system that incorporates environmental factors needs healthcare, social care and non‐health data linkage at individual and small‐area levels. Our objective was to establish privacy‐preserving household record linkage for England to ensure person‐level data remain secure and private when linked with data from households or the wider environment.

**Methods:**

A stakeholder workshop with participants from our regional health board, together with the regional data processor, and the national data provider. The workshop discussed the risks and benefits of household linkages. This group then co‐designed actionable dataflows between national and local data controllers and processors.

**Results:**

A process was defined whereby the Personal Demographics Service, which includes the addresses of all patients of the National Health Service (NHS) in England, was used to match patients to a home identifier, for the time they are recorded as living at that address. Discussions with NHS England resulted in secure and quality‐assured data linkages and a plan to flow these pseudonymised data onwards into regional health boards. Methods were established, including the generation of matching algorithms, transfer processes and information governance approvals. Our collaboration accelerated the development of a new data governance application, facilitating future public health intervention evaluations.

**Conclusion:**

These activities have established a secure method for protecting the privacy of NHS patients in England, while allowing linkage of wider environmental data. This enables local health systems to learn from their data and improve health by optimizing non‐health factors. Proportionate governance of health and linked non‐health data is practical in England for incorporating key environmental factors into a learning health system.

## 1 BACKGROUND

Learning Health Systems have been described as “system evaluations designed to achieve continuous rapid improvement in health and healthcare and to transform organizational practice.”^1^ This framework usually focuses on healthcare providers and patient outcomes. Here we apply this framework to the context in England where social care, local government and other kinds of organizations work with the National Health Service (NHS) in a learning health system called an Integrated Care System, to improve population health, via actions on modifiable environmental determinants such as housing, transportation and public recreation spaces. Local health systems in England wish to prioritize prevention following the Hewitt Review calling for more emphasis on prevention.^2^, ^3^, ^4^ As part of this system, each of the 42 regional Integrated Care Boards (ICB) need non‐NHS‐sourced environmental and social data to help set and monitor policies for preventive measures. Innovative system evolution will be needed to allow quality evidence generation to guide optimal public health initiatives, including linking data from sources such as social care and environmental features in a population health learning system. Public health experts may enrich the work of the local health systems given securely held linked cross‐sectoral data sets. These data sets are crucial for understanding how policy, practice and society can be organized to reduce ill health and prevent those with the least resources from having the greatest burden of ill health, often described as social determinants of health.^5^, ^6^

Interventions in non‐health systems, such as housing improvements, can require long follow‐up periods to evaluate their impact. In these cases, data that have been aggregated into small areas may be unsuitable for intervention evaluation due to changes in the underlying population,^7^, ^8^ where healthier (or less healthy) people may have displaced the original population, producing false signals of beneficial (or harmful) effects of the intervention. Changes in the underlying population are particularly likely if the intervention is intended to improve an area, such as creating local parks and paths along waterways, resulting in gentrification.^9^, ^10^ Using individual‐level data for the resident population with links to their health conditions from before, during and after an intervention ensures evidence is more robust. Generating datasets using routinely collected data where the environmental exposures precede the outcomes is possible using household linkages with dates of residence. Further benefits of household linkages include: grouping people into homes to investigate household income impacts on health; the impact of chronic health conditions on other residents, including children; and uncovering patterns of communicable disease transmission in households, which is critical for pandemic planning.

Whole System data linkage endeavours with linked social determinants of health and, in particular, environmental data, which exist in few countries, namely Canada, Scotland, Norway, Australia, Wales and some parts of England.^11^, ^12^, ^13^, ^14^, ^15^ The impact of these linked data sets is demonstrable in, for example, Wales where longitudinal household linkages in the Secure Anonymised Information Linkage Databank provided evidence of reductions in emergency hospital admissions over a decade for residents whose homes were updated to a national quality standard,^8^ and the importance of planning sufficient green space, preventing the onset of common mental health conditions requiring treatment in primary care, particularly for those in deprived areas.^16^, ^17^ This underscores the importance of data linkage methodologies to understand the interaction between environmental factors and health outcomes, with all focussed on informing evidence‐based policymaking.^18^

England is the only nation in the United Kingdom without a consistent national residential linkage method in place, for the addition of granular environmental data.^7^, ^19^, ^20^, ^21^, ^22^ Opportunity costs from not enabling these linkages are large versus the benefits of better decision‐making from more insightful data. Here, we (GroundsWell, an academic research consortium) share our experiences of working with partners in NHS England to put in place a mechanism for linking people's health records to their household‐level exposures to provide evidence to change policy, preventing ill health. Our aim was to establish privacy‐preserving household record linkage for England to ensure person‐level data remain secure and private when linked with data from households or the wider environment. We use the potential to link environmental data for green and blue spaces to health outcomes as a test case for an England‐wide environmental determinant‐enabled learning health system.

## 2 METHODS

This work focussed on working with 1 of the 42 regional ICBs in England, who together provide health care for the NHS across the 57 million people in England. Identifiable data provided by NHS England for use by the ICB are held and processed by its Data Services for Commissioners Regional Offices (DSCRO). For the remainder of this article, NHS England is the data provider (providing a flow of data to the DSCRO containing the Unique Property Reference Numbers [UPRNs, a unique, linkable identifier for every spatial address in the United Kingdom, which is consistent across time for each home address^23^] linked to patient identifiers); the DSCRO is the data processor (hosting and anonymising the data on behalf of the ICB); and the regional ICB and GroundsWell consortium team facilitate the flow and use of the household‐level data.

**FIGURE 1:**
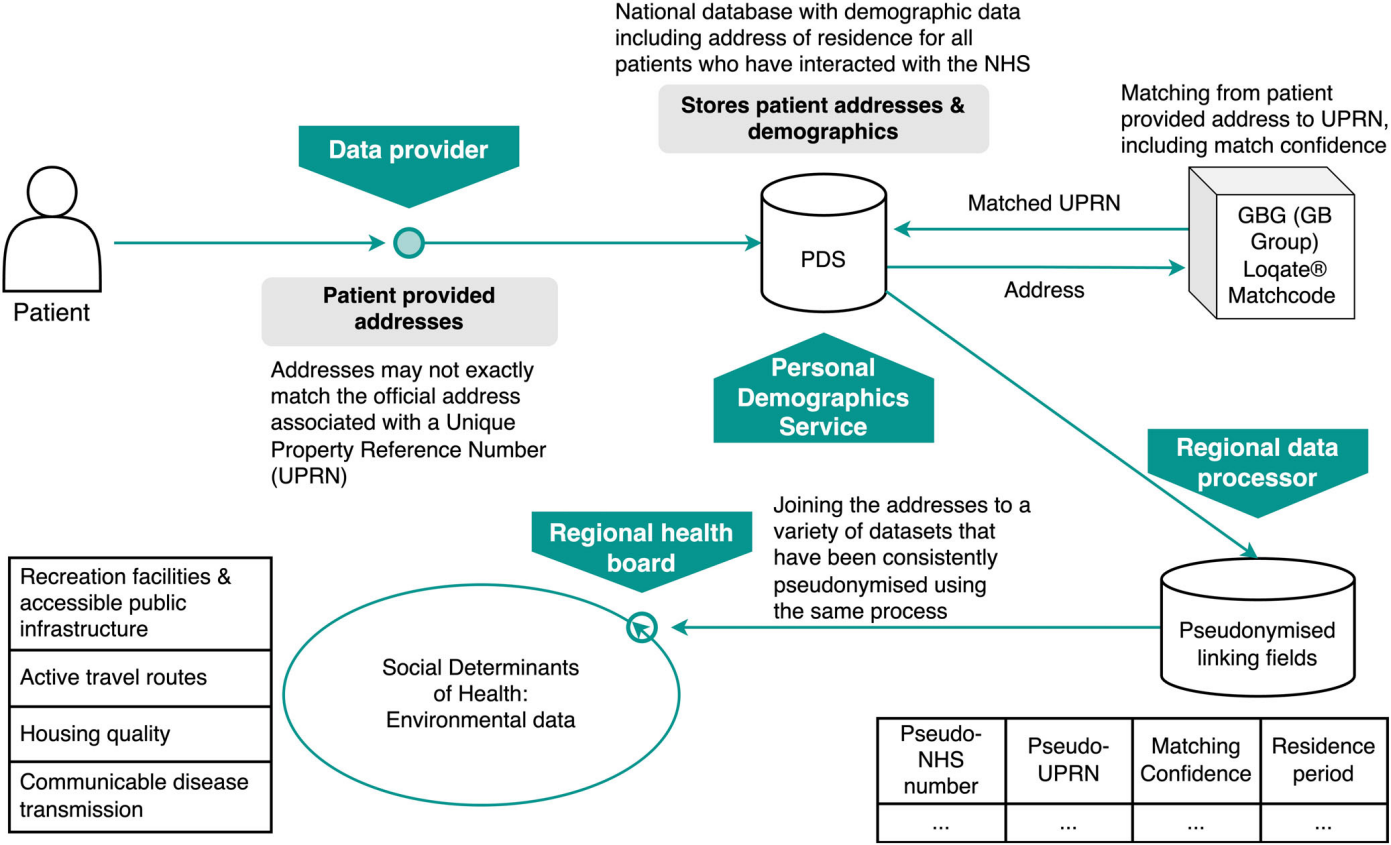
Household linkage data flow process for creating a public health learning system.

The data provider manages the Personal Demographics Service (PDS), the definitive source of demographic data for NHS patients in England.^24^ A key component of the PDS is the patient‐provided addresses, which may not exactly match the official address as held by the UK Royal Mail's Postcode Address File (PAF).^25^ It is these official addresses that are used to map to each home using the UPRN. The data processor has undertaken work to map the patient‐provided addresses to their official address and corresponding UPRN. This mapping was done using the commercial product GBG Loqate® Matchcode tool.^26^ Using UPRNs makes it possible to group individuals into households and enable linkage to external information such as distance to local health services, outside air quality or how much green space is nearby. The process of data linkage and data flow to the regional ICB is detailed in Figure 1.

### 2.1 Data access request

The GroundsWell consortium used urban green and blue spaces as an exemplar of a social determinant of health important for communities at high risk of non‐communicable diseases (NCD).^27^ We were commissioned by our local government and an ICB to evaluate health outcomes linked to green and blue spaces.

In the United Kingdom, we follow the European General Data Protection Regulations (GDPR). The Data Protection Act (2018) implements the GDPR. The GDPR relates to any personal data that identify individuals. For research purposes, we request access to data that are held securely within the NHS systems, which are patient‐level records with all identifiers (e.g., names, addresses) removed or replaced. Data that are fully anonymised or aggregated data are *not* regulated by the GDPR or DPA2018, providing the anonymisation or aggregation has not been done in a reversible way.^28^

We applied to the ICB data governance group for access to pseudonymised individual record health data, with linkage to household‐related data for named GroundsWell researchers who had undertaken training to qualify to access securely held pseudonymised individual‐level data. These data had all names, addresses and other identifying information removed, replaced with unique and consistently allocated linking fields for individuals, enabling data to be joined for the same person across different health and care datasets while protecting privacy. Once pseudonymised, each patient record retains an indication of their home neighbourhood (Lower layer Super Output Area).

### 2.2 Knowledge exchange workshop

We convened a stakeholder workshop in April 2023 to initiate proceedings to move towards a household data linkage system. The workshop included representatives from the health service, local government and the GroundsWell consortium. During the workshop, GroundsWell shared household data linkage successes from other UK nations and facilitated a discussion on (i) how GroundsWell would use linked data and the impact these population health evaluations could have on policy and practice; (ii) the benefits and risks of household data linkages; and (iii) the processes needed to allow these types of population health evaluations.

### 2.3 Ongoing partnership working

Following the workshop, to develop the household linkages, we began regular meetings with the data provider to establish the format of the required data flow. We also began meeting with the data processor, to ensure that they were able to receive the data according to their regional IG needs.

### 2.4 Ethics statement

We set out to assess and improve household data linkages in a specific region, and not to produce generalisable knowledge. As such, this work constituted operational improvement activities that are exempt from ethics review. The primary purpose of this report is to share lessons learnt from partnership working across NHS bodies and with academics, and to discuss implications for addressing other public and preventive health challenges using household data linkages as the bedrock of a public health learning system. The funders had no role in the design of this partnership working process, decision to publish or preparation of the manuscript.

## 3 RESULTS

### 3.1 Knowledge exchange workshop

During the workshop, NHS partners raised topics that required further consideration, including potential re‐identification of patients or their locations due to unique combinations across the number of different health and environmental variables. We reflected on the need for granular data to uncover patterns and generate evidence to guide policy effectively to improve health. Of note was the need for longitudinal address histories to account for the effect of gentrification when evaluating interventions. We also discussed the opportunity costs to public health and well‐being from delays in accessing data, or barriers to linking data, in terms of lack of evidence to guide policy and practice.^29^

The outcome was a co‐designed plan to work with a national data provider to integrate data systems and processes to enable household linkage. This plan would work across the footprint of a regional ICB that would meet the needs identified by participants at the knowledge exchange workshop. We reflect here that only when different stakeholders came together, were the challenges of establishing a national mechanism for household data linkage thoroughly discussed through different lenses. This resulted in balancing the benefits with the risks and measurements needed, so ultimately patients can benefit from the actions taken by those representing their interests.^30^

### 3.2 Data provider meetings

**FIGURE 2:**
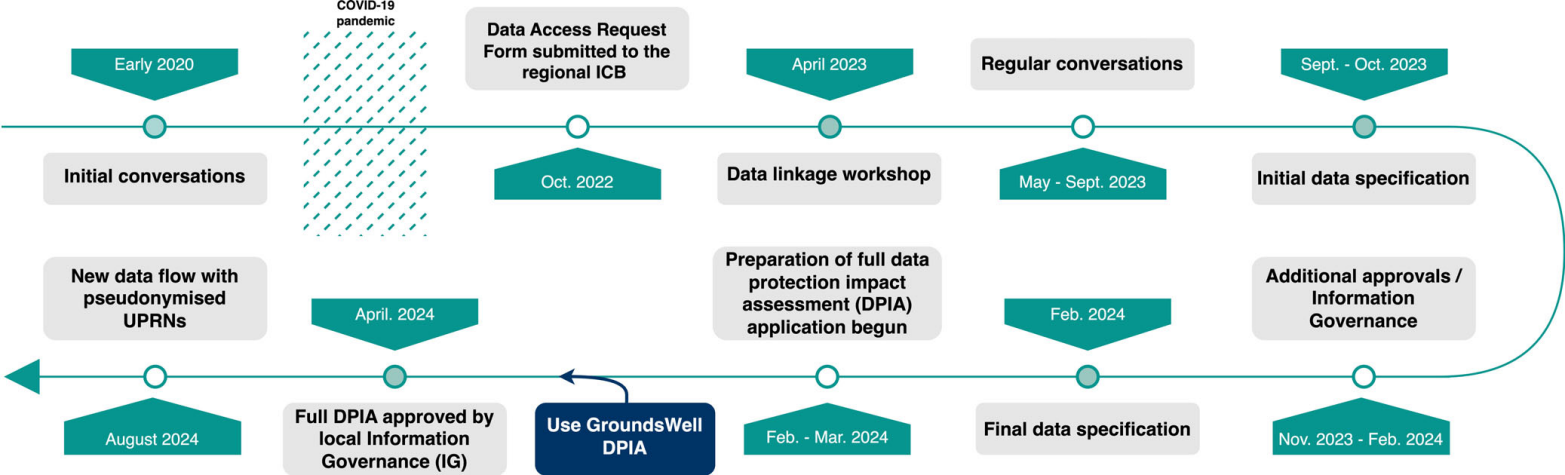
Timeline of stakeholder engagement and partnership working to progress household data linkages in England.

Following the knowledge exchange workshop, we held 11 meetings with a senior data engineer at the data provider to develop the household linkages, as frequently as every 2 weeks, from May 2023 to February 2024 (Figure 2). During these meetings, we learned that the data provider had decided to add a UPRN field to the PDS dataset. This UPRN field will be populated (and validated) when an address is added or updated, as people move home; thus, it may take several years for significant numbers of addresses to be assigned a UPRN using this method alone. To have a population‐wide household‐linked dataset there is still, therefore, the need to retrospectively match existing, non‐validated, patient‐provided addresses in the PDS to an official address and onwards to a UPRN.

For the public health learning system to meet the needs identified by participants at the knowledge exchange workshop, we co‐developed the minimum number of fields to flow from the data provider to the processor. This included: the NHS identification number of the patient, the UPRN of the patient's home; the parent UPRN which would be present if a property is part of a larger property (e.g., an apartment building); and the length of time the patient has lived at each home. We also ensured that a series of matching metrics would be part of the data flow to give an indication of the confidence of the match from the patient‐provided address to the official address associated with a UPRN (Figure 1). Matching confidence metrics were discussed at the workshop as essential to assess the quality of the matches for different evaluations. For intervention‐based evaluations, having an indication of linkage accuracy between person and location is key, because a home improvement (for example) will only impact those people within the property itself, rather than everyone on the same street or apartment building.

Progress in operationalizing the household linkages was slowed because the senior data engineer was frequently required to work on national health protection projects, such as those monitoring flu vaccinations. By February 2024, the data were ready to flow from the data provider to the data processor, subject to the relevant IG being satisfied.

### 3.3 Data processor meetings

In our continued work with the data processor, we took advice from their IG lead that further regional IG approval would be required to make the data available to the ICB. In response, we co‐created a data specification of the data anticipated to flow from data provider to the data processor, with accompanying timelines. This ensured that the data pseudonymisation requirements of the processor were explicitly stated, with time planned to complete processing once the data were received, reflecting this in the accompanying timelines. In September 2023, the initial data specification was approved, in principle, by the data processor's regional IG committee. The final data specification, updated to match the final agreed data flow, was approved in February 2024 (Figure 2).

### 3.4 Data provider information governance

In Winter 2023, the senior data engineer at the data provider advised that we may need further IG approval from the data provider before flowing data to the processor, and subsequently to our regional ICB. The senior data engineer had initially corresponded with their colleagues at the data provider about the IG approval request. We subsequently invited the IG lead at the data processor to attend our meetings. In early 2024, there was correspondence between the IG leads at the data processor and the data provider. Following this example of partnership working between the data provider and the processor, we were advised by the data provider (in February 2024) that a full governance application (a Data Protection Impact Assessment—DPIA) would be needed to mitigate the risks of identifying patients. The governance application involves a thorough check of all data flows and intended uses to be provided by the team, for national experts in IG to assess the risk of identification of a patient against the benefits of using this newly derived, linked dataset. Our DPIA was approved in April 2024. This covered the flow of UPRN‐based pseudonymised households, with nested patient NHS numbers, to the ICB. The permissions to link external data at UPRN level is currently being assessed by the national data provider.

### 3.5 Data flow status

As of August 2024, the pseudonymised UPRNs are available to the ICB from the data processor. Moreover, since the data processor provides services for multiple ICBs, the pseudonymised UPRNs have been made available to all ICB clients. In addition, the pseudonymised UPRNs will soon be made available to all data processors in England, paving the way for all 57 million registered patients in England to be included in studies investigating the social and environmental determinants of health using household linkages.

## 4 DISCUSSION

Household‐level data linkage is a vital part of a learning health system to improve the health of current and future generations, particularly through informing joint planning and public health place‐based interventions. We have highlighted the substantial time and resources from multiple stakeholders required to embed partnership working across the health data ecosystem to co‐develop household data linkages creating a population health learning system. Time was needed for both the information governance (IG) permissions and the technical specification. Time and resource allocations are sensitive to the, sometimes different, perspectives of data providers and data users. Different perspectives required discussion, and justification of the need for population health management and the development of feedback processes and documentation.

As of August 2024, this complex process has taken 22 months from our initial application for access to data, including time spent developing a shared understanding and purpose, regional data governance processes at the ICB, two submissions to the governance panels at the data processor, co‐creating a workable data specification for data flow from the provider to the processor and operationalizing the linkages (see Figure 2 for summary). This excludes the additional delay due to the pandemic, which occurred during the early stages of this process.

Time is required not just for workshops, meetings and technical programming, but for those employed in the NHS and local government to respond to emerging situations such as seasonal flu vaccinations and measles outbreaks. Although the NHS has increased the prevention of ill health in priority, initiatives enabling NHS staff to dedicate time to work with a range of stakeholders, building a multidisciplinary team, requires more effort than has so far been resourced.

Different perspectives were evident in the initial knowledge exchange workshop, with some “benefits” of household data linkages identified by academics and local government also identified as “risks” by data providers. The use of language sometimes seemed to alter depending on the role of the data provider or data user, and the subject being discussed. This illustrates the importance of open, non‐judgmental discussions, and working towards a shared inclusive language.^31^

Many organizations have sought to create data linkage systems within local government organizations who provide social care and non‐health data. In Devon, England, timelines were comparable and extensive,^15^ and efforts like Connected Bradford have shown the potential for success in privacy‐preserving record linkage in England with a singularly dominated health data ecosystem.^14^, ^15^ However, these instances are rare, requiring concerted efforts to develop shared language, understanding and priorities, and can be impeded by the complexity of merging data across diverse systems.

The success stories from discrete regions in England, where household linkages have been established, illuminate one path forward but also emphasize the need for systemic change to facilitate the widespread adoption of these practices in England. Establishing a household‐level data linkage framework, like the approach in Wales developed by Rodgers and colleagues,^32^, ^33^ needs alignment on address matching methodologies, or using a consistently matched central source (such as the one generated by NHS England). Otherwise, different methodologies will introduce artefacts in the linkages, becoming apparent when household linkage analyses are carried out across multiple regions. Multiple region analyses are on the horizon with the advent of England's subnational Secure Data Environments (SDEs), which aim to enable federated population health research. The approach we took, working with the national data provider from the start, facilitates this national approach to household linkages.

The address matching service used by the data provider is a commercial product, and while a match score is provided as part of the service, the matching algorithm is not in the public domain. This black box approach potentially introduces biases into the linkage which will be opaque to the users of the data and will incur an ongoing cost. Comparison with other, openly available, address matching algorithms would ensure that the linkage described here is robust.

An additional reflection from this work is the difficulty in finding an official ‘front door’ into some organizations. This has resulted in relying on networks to find relevant people to work with, often on a best‐efforts basis. This contributed to us inadvertently pursuing a route where the full IG framework was not apparent from the outset. One systems level change would be to have a more formal route into these organizations to enable multidisciplinary partnership working to a set framework, recognizing that with additional formality there will be less flexibility. We did not vigorously pursue an early suggestion by the data provider to embed university researchers into the main data provider (NHS England). While the level of effort to achieve IG clearance to allow researcher access to the address data would have been high, it is possible that a secondment, under strict direction, would have yielded a net gain in the time taken to achieve this work, while resulting in more accessible robust documentation of the data generated.

The UPRN is a publicly available immutable reference which is equivalent to raw address text. While it is assumed that the use of the UPRN would have controls such as pseudonymisation, there is a residual risk of re‐identification to be managed. For instance, inferences based on counts of residents in each property may identify care homes or student accommodation. While individual data controllers who enable the use of UPRNs will likely have in place local risk mitigation strategies, there is not a common set of best practices. As the use of UPRNs in a public health learning system increases, there is the need to develop common statistical disclosure control methodologies to ensure privacy is preserved.

### 4.1 Recommendations

Advancing UK health and related data governance requires many simultaneous changes. We have demonstrated the advances made when key senior stakeholders come together and talk in common non‐discipline and non‐organization language or jargon. Recommendations for moving forwards include:

1. Establish formal routes to communicate with relevant organizations, enabling linkage bias evaluation and tailored data quality checks.^34^, ^35^
2. Embed researchers within data providers, enabling robust linkages and streamlined communication.
3. Develop an understanding of the disclosure risks and mitigations (including statistical disclosure control) for using these linked data sets.
4. Establish a route for easy adoption of the work described here for all ICBs in England.
5. Involve the public in further discussions and dissemination to maintain the use of inclusive language and encourage wider engagement by all stakeholders.
6. Agree common address matching methodologies across the United Kingdom.^33^, ^36^, ^37^

A coordinated community of practice, bringing together representation from the national data provider, data processors, regional ICBs, academics and local government should be established to fulfil these recommendations. Regionally, public voices should be given power in these discussions as part of streamlined governance.

### 4.2 Impact

While we do not claim that we are the sole reason household linkages using UPRNs are being rolled out across England, we note that we: launched the process locally on behalf of an ICB, developed a relationship with the data provider, made the case for longitudinal household linkages to the data provider, co‐designed the format of the data flow, brokered the relationship between the data provider and processor, and co‐developed IG documentation for use locally. These steps, including our resulting DPIA, are being used by IG experts in many other ICBs throughout England. Our process means that others will not need to go through the full process, dramatically reducing the time required to access UPRN‐level household‐linked data. We are happy to share relevant parts of our documentation (e.g., DPIA, data specification, etc.), upon request to the corresponding author.

### 4.3 Future steps

A UPRN‐based household‐linked dataset is now available within our regional ICB, and we will develop mechanisms to assess the quality of the linkages to check for systemic biases. We are refining the linkage process to bring wider environmental measures to users of the data, ensuring patient privacy is protected. It is our aim for these data to be accessed by NHS‐academic teams across England to evaluate the impact of place and identify modifiable factors to reduce the burden of ill health for the population living in 45 million addresses in England.

We continue to work with key senior stakeholders, demonstrating the population health benefits of longitudinal household data linkages, with methods to minimize risks of identification. We are building collaborations with data providers by encouraging partnership working in England, enabling further linkages of environmental and health data for longitudinal analyses. In the initial steps towards creating a public health learning system documented here, the data provider was NHS England, because they hold a national database of addresses data for NHS patients in England, the PDS. In future, other organizations may be the data provider for new household indicators. For example, local government providing distances to the nearest park and details of new developments planned, or social housing providers, sending housing quality data for all homes. The process developed here means new data providers may send their anonymised household indicator‐UPRN datasets to the data processor, where a copy of the identifiable home‐patient (UPRN‐NHS number) data is maintained. Completing these linkages should be subject to governance approvals that include checks for potential uniqueness, ensuring no individuals may be identified—essential for data governance approvals.

## 5 CONCLUSION

The technology and cultural shifts needed to sustain learning cycles that consider environmental determinants of health and care are advancing in England through building Integrated Care Systems/Boards. Household‐level data linkage, which is key to this work, has been inconsistent across regions in England but is well established in Scotland and Wales. We have co‐developed a proportionate governance framework to allow household‐level health and related data linkage across England and started to implement this with NHS national data service colleagues in an English Integrated Care System. The linked data will enable new insights to inform environmental actions, for example, housing improvements, which are vital for continuous, locally sensitive learning on how to improve population health and reduce inequalities.

## AUTHOR CONTRIBUTIONS

All co‐authors substantially contributed to the conception or design of the work or the writing, and/or to significant review of this manuscript, and all who qualify for authorship are listed as co‐authors.

## CONFLICT OF INTEREST STATEMENT

The authors declare no conflicts of interest.
